# Approaching “Phantom Heritability” in Psychiatry by Hypothesis-Driven Gene–Gene Interactions

**DOI:** 10.3389/fnhum.2013.00210

**Published:** 2013-05-16

**Authors:** Diego Luiz Rovaris, Nina Roth Mota, Sidia Maria Callegari-Jacques, Claiton Henrique Dotto Bau

**Affiliations:** ^1^Department of Genetics, Instituto de Biociências, Universidade Federal do Rio Grande do SulPorto Alegre, Brazil; ^2^Department of Statistics, Instituto de Matemática, Universidade Federal do Rio Grande do SulPorto Alegre, Brazil

The so called “phantom heritability” has being haunting the scientific community for quite some time and it is now subject of intense debate. Zuk et al. (2012) acknowledged that even if we identify all the genetic variants related to any trait, full understanding of its heritability will not be conquered if we do not face up to the epistatic effects. The authors pointed out that when estimating the explained additive heritability, obtained by $\pi_{\text{explained}}=\text{additive }{\text{h}}_{\text{known}}^{2}/\text{additive }{\text{h}}_{\text{all}}^{2}$, the denominator is estimated indirectly from phenotypic correlations in a population (apparent heritability, ${\text{h}}_{\text{pop}}^{\text{2}}$). In this sense, the “missing heritability” (1−π_explained_) is calculated by assuming that ${\text{h}}_{\text{all}}^{\text{2}}={\text{h}}_{\text{pop}}^{\text{2}}$. However, for traits with genetic and/or gene-environment interactions (which applies to practically all psychiatric phenotypes), ${\text{h}}_{\text{pop}}^{\text{2}}$ significantly exceeds ${\text{h}}_{\text{all}}^{\text{2}}$. In this case, even when all variants that contribute to the phenotype are identified, the missing heritability will not be zero and will converge to $1-{\text{h}}_{\text{all}}^{\text{2}}/{\text{h}}_{\text{pop}}^{\text{2}}$, phenomenon called “phantom heritability.”

The role of gene–gene interactions in several psychiatric phenotypes is well recognized, but the biological underpinnings of this concept have not been sufficiently discussed. We suggest that a major limitation involves the lack of data-driven hypotheses for the study of such epistatic effects. While hypothesis-free whole genome epistatic analyses would require an unrealistic sample size, hypothesis-driven gene–gene interaction studies require a reduced number of multiple tests and might help reducing the “phantom heritability.”

Several statistical methods for accessing epistatic effects in genome-wide association studies (GWAS) have been created or improved in the last years (Cowper-Sal lari et al., 2011; Gyenesei et al., 2012; Pandey et al., 2012; Piriyapongsa et al., 2012; Ma et al., 2013; Pendergrass et al., 2013), but they still fail to provide biological interpretation. One option for this problem is to only test interactions with biological plausibility by implementing semi-exhaustive approaches in GWAS using experimental knowledge of biological networks. By applying this method to the Wellcome Trust Case-Control Consortium data sets, Emily et al. (2009) reduced the number of SNPs pairs inserted in the analysis (1.25 × 10^11^ to 3.5 × 10^6^) and reported a significant case of epistasis between *PDGFR-B* and *KITLG* genes in susceptibility to bipolar disorder. However, this finding was not replicated yet and it is not supported by experimental data.

On the other hand, *in vitro* and *in vivo* experimental assays, as well as protein modeling and bioinformatic analyses of protein–protein interface are exciting sources of plausible hypothesis for the study of gene–gene interactions in candidate gene association studies. Excellent examples are given by Gonzalez et al. (2012) and Borroto-Escuela et al. (2011). These authors evaluated the heteromerization patterns between two dopamine receptors (DRD2 and DRD4) according to their different isoforms and were able to show that common variants may modulate this process. Based on these data, Mota et al. (2013) have demonstrated, in two independent samples, a significant epistatic effect on the susceptibility of alcohol dependence that may likely reflect the different heteromerization patterns previously described. Similarly, mineralocorticoid and glucocorticoid receptors (MR and GR) are physically interacting proteins with well characterized functional polymorphisms in their coding genes (*NR3C2* and *NR3C1*, respectively). A MR-GR epistatic effect on nicotine dependence susceptibility (Rovaris et al., 2013) is consistent with the findings of differential heteromerization according to the expression of GR-beta isoform, which is modified by the genetic variation examined.

**Figure 1:**
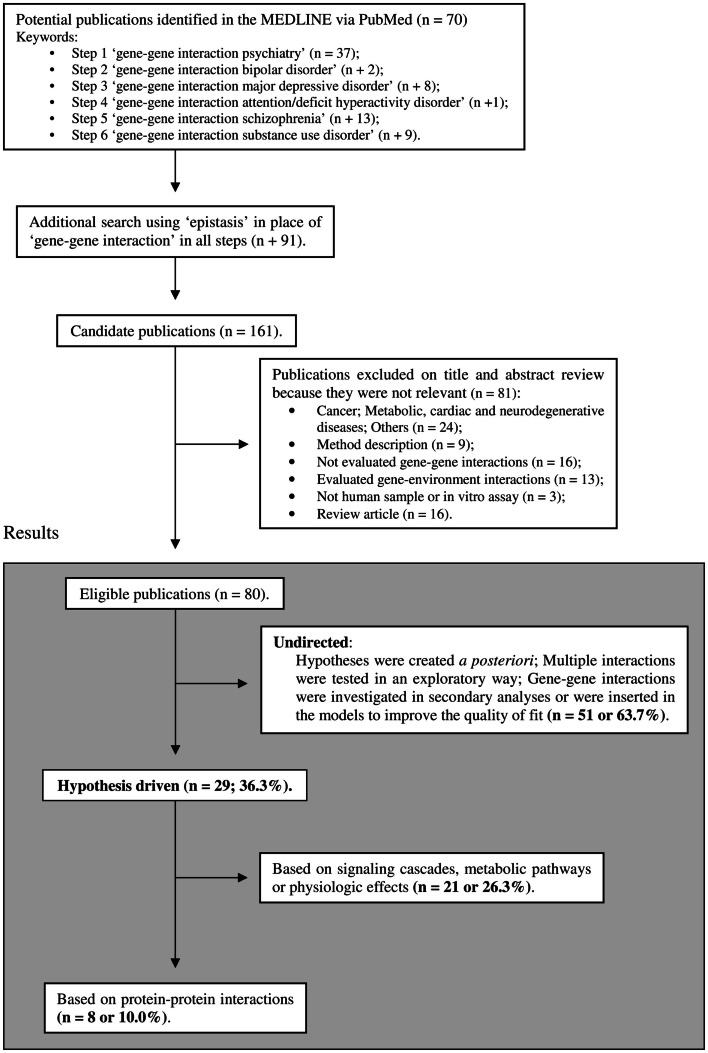
**Selection of studies included in the review**. The limits were: publication in the last 5 years (published until January, 2013), humans, English language, full text available, and MEDLINE. The list of included articles is available upon request.

Despite hypothesis-driven strategies being widely accepted and their advantages well recognized, few empirical studies have applied this approach to address gene–gene interactions. In an intent to clarify the extent to which this approach is neglected in psychiatric genetic studies, we conducted a search on recent MEDLINE-indexed literature (inclusion criteria are shown in Figure 1). This search was designed to sample relevant psychiatric phenotypes and was limited to the last 5 years, period where both GWAS data and functional evidence on genetic polymorphisms became widely available. Although thousands of molecular genetics studies on psychiatric disorders have been published, only 80 gene–gene interaction reports were eligible for analyses according to our inclusion criteria. From the selected 80 studies, 29 (∼36%) tested interactions from *a priori* hypotheses and 8 (10%) used data on protein–protein physical interactions (Figure 1). These results suggest that hypothesis-driven gene–gene interaction studies remain a somewhat neglected in psychiatric genetic studies, despite their well recognized potential to increase the explained heritability of psychiatric traits.

The evaluation of hypothesis-driven gene–gene interactions in candidate gene association studies is a novel approach that should be increasingly pursued in neuroscience and psychiatric genetic studies. In this sense, this line of research could even help in the development and improvement of techniques (machine learning methods, for example) to be applied in GWAS analyses of gene–gene interactions. Some approaches have recently been implemented with this objective in cancer biology and cardiology (Andrew et al., 2012; Ma et al., 2012). Interrelationships of computational statistics and modern molecular biology could help narrow the gap between “missing” and “phantom” heritabilities in psychiatric genetics.
